# Supplementary material for: Benchmarking distributed data warehouse solutions for storing genomic variant information
Source: Database (Oxford). 2017 Jul 11;2017:bax049. doi: 10.1093/database/bax049 (PMC5504537; doi:10.1093/database/bax049)
Supplement: Supplementary Data [file bax049_Supp.pdf]

Benchmarking distributed data warehouse solutions for storing genomic variant information – *version with tracked changes*

Marek S. Wiewiórka[1], Dawid P. Wysakowicz[1], Michał J. Okoniewski[2] and Tomasz Gambin *[1,3]

[1]Institute of Computer Science, Warsaw University of Technology, Warsaw, 00-665, Poland

[2]Scientific IT Services, ETH Zurich, Zurich, 8092, Switzerland

[3]Department of Medical Genetics, Institute of Mother and Child, Warsaw, 01-211, Poland

March 31, 2017

Abstract

Motivation: Genomic-based personalised medicine encompasses storing, analysing and interpreting genomic variants as its central issues. At a time when thousands of patients' sequenced exomes and genomes are becoming available, there is a growing need for efficient database storage and querying. The answer could be the application of modern distributed storage systems and query engines. However, the application of large genomic variant databases to this problem have not been sufficiently far explored so far in the literature.

Methods: To investigate the effectiveness of modern columnar storage (column-oriented DBMS) and query engines, we have developed a prototypic genomic variant data warehouse, populated with large generated content of genomic variants and phenotypic data. Next, we have benchmarked performance of a number of combinations of distributed storages and query engines on a set of SQL queries that address biological questions essential for both research and medical applications. In addition, a non-distributed, analytical database (MonetDB) has been used as a baseline.

Results and Discussion: Comparison of query execution times confirm that distributed data warehousing solutions outperform classic relational database man-

[0]Corresponding author: tgambin@ii.pw.edu.pl

agement systems. Moreover, pre-aggregation and further denormalization of data, which reduce the number of distributed join operations, significantly improve query performance by several orders of magnitude. Most of distributed back-ends offer a good performance for complex analytical queries, while the ORC format paired with Presto and Parquet with Spark 2 query engines provide, on average, the lowest execution times. Apache Kudu on the other hand, is the only solution that guarantees a sub-second performance for simple genome range queries returning a small subset of data, where low-latency response is expected, while still offering decent performance for running analytical queries. In summary, research and clinical applications that require the storage and analysis of variants from thousands of samples can benefit from the scalability and performance of distributed data warehouse solutions.

Supplementary information: The detailed description of the project can be found at `https://github.com/ZSI-Bio/variantsdwh`

1 INTRODUCTION

1.1 Variant information in genomic-based personalised medicine and biomedical research

In the current era of high-throughput sequencing, the reliable and comprehensive analysis of genomic variant data have become a central task in many clinical and research applications related to precision ~~personalised~~ medicine. Joint analysis of such data from thousands of samples collected under large scale sequencing projects, such as Exome Sequencing Project (ESP, `http://evs.gs.washington.edu/EVS/`), The Atherosclerosis Risk in Communities Study (ARIC) [1], Centers for Mendelian Genomics (CMG) [2], UK10K [3], The Cancer Genome Atlas (TCGA) [4]) provides a detailed and medically useful insight into molecular basis of many genetic conditions.

Although a plethora of statistical methods (e.g. for variant prioritization [5, 6, 7, 8, 9] or variant association [10, 11, 12, 13, 14] was developed, there is a lack of tools that allow researchers to perform *ad hoc*, unrestricted queries on large data sets. Such tools should be powerful enough to deal with population-scale sequencing data that will be generated by such large-scale projects as Genomic England's "100,000 Genomes Project" (`http://www.genomicsengland.co.uk/the-100000-genomes-project/`) or Precision Medicine Initiative (PMI, `http://www.nih.gov/precision-medicine-initiative-cohort-program`) announced by the US administration, which aim in sequencing of at least 1 million Americans.

* ~~Corresponding author: tgambin@ii.pw.edu.pl~~

The early attempts of applying big data technologies to interactive analyses of sequencing datasets were focused on providing the end user with an API (*Application Programming Interface*) in Pig [15] or Scala [16] languages and integration with the exiting bioinformatics file formats using middleware libraries like Hadoop-BAM [17]. Those approaches while very flexible, but impose imperative programming paradigm which assume that the end user explicitly declares query execution plans. This is not suitable for many researchers and data scientists who are not experts in distributed computing programming at the same time.

Recently, several emerging technologies such as big data query engines offering SQL (*Structured Query Language*) interfaces like Apache Impala (`http://impala.io/`), Apache SparkSQL (`https://spark.apache.org/`), Presto (`https://prestodb.io/`), Apache Drill (`https://drill.apache.org/`) made it possible to adapt declarative paradigms of programming to analysing very large datasets. Finally, big data multi-dimensional analytics cube solutions such as Apache Kylin (`http://kylin.apache.org/`) can significantly speed up SQL queries by storing already pre-aggregated results in a fast noSQL database (e.g. Apache HBase).

Efficient *ad hoc* data analysis has been already for years a main goal of OLAP (*Online Analytical Processing*) solutions design based upon data warehouses. In the case of genomic OLAP systems, the end-users are clinicians and medical researchers. Both groups have different needs and expectations towards data analysis, still those are not mutually exclusive. On the clinical level it is important to find knowledge about variants in well-known genes and to compare the variant information of newly sequenced patients against a larger knowledge base. Here the *ad hoc* question may have the form e.g. ”tell me what diseases and phenotypes may have a patient having a specific set of variants”. On the research side, the queries are focused on unsupervised searches that combine sets of variants, sets of genomic intervals and phenotypic information. This is often about getting the estimates of specific measures of aggregates, e.g. ”tell me which phenotype has its related genes with an over-represented amount of variants”.

Current solutions for analyzing sequencing data using SQL and storing genomic variant information can be divided into two categories. First group of tools tries to take advantage of classic single-node RDBMS (*Relational Database Management System*) like MySQL [18, 19] or newer analytical with column-oriented store like MonetDB [20]. Those solutions are able to provide flexibility and very good ANSI SQL conformance and reasonable performance ([21] while working on datasets that are already pre-aggregated (e.g. using Apache Pig in case of [19]) or limited in size (500MB reported by [20]) . On the other hand, they do not offer horizontal scalability, efficiently compressed store and high availability features out of the box.

The other group of prototypes focuses on providing distributed and scalable bioinformatics file formats equivalent to well known ones such as Binary Alignment Map (BAM)

or Variant Calling Format (VCF). The major example of such an approach is ADAM (*Avro Data Alignment Map*) formats family [22] using Avro (`https://avro.apache.org/`) data serialization and Parquet (`https://parquet.apache.org/`) columnar data representation. These files can be processed using popular query engines like Apache Impala, Apache Spark or Presto. Such a modular approach where storage is not tightly connected to a specific computing framework opens up possibility of choosing freely both a query engine and file format that provides the best performance.

The goal of this prototyping study is to provide hints on combining both approaches in order to create scalable and flexible data warehouse infrastructures for genomic population-scale studies at single sample and variant granularity. For this purposes, the benchmarking suite consisting of data generator, data model and set of queries has been proposed. The benchmarking results of this project are intended to point out the future directions of work on genomic variant data warehouse biobanks. Such database study may be needed in all the areas of application of genomic systems: research, medical and commercial.

1.2 Biomedical issues that require *ad hoc* variant data analysis

Accurate detection and interpretation of genomic variation obtained from NGS data became the central issues in the ~~personalized~~ precision medicine and human genetics research studies. To enable a comprehensive and efficient variant analysis the storage and query engines should allow to run a wide range of *ad hoc* queries providing the answer to the most relevant biomedical questions.

Variant prioritization Although many methods have been proposed to evaluate variant pathogenicity [23], the allele frequency measured in control populations remains as one of the best variant effect predictors [24]. Most of the disease-causing variants are absent or rarely observed in general population but their allele frequency may vary among ethnic groups. Therefore, it is important to distinguish population-specific polymorphisms (likely benign) from real pathogenic variants that are rare enough in every sub-population represented in the control data.

Publicly available databases (1000genomes, ESP, ExAC) report variant frequencies for a small set of pre-defined ethnic groups (e.g. Europeans, Africans, Asians). Still, no information about variant frequencies in smaller sub-populations (e.g. on the country/state/county level) can be found in them. There are many reasons of not reporting this potentially useful data. First, a detailed information about origin of samples included in these studies was not always collected or was not available. Second, the number of individuals in other sub-populations was too small so reporting allele frequency was not justified. Finally, storing allele frequency in the VCF file for every possible level of granularity of population structure would become impractical.

It can be expected that first two issues will be resolved in the near future when more

good quality data sets will become available. Still, the pre-computing, updating and storing of such high dimensional allele frequency information will remain a challenge. While testing the prototype, the main aspect taken into account was the performance of our variant data warehouse in calculating variants' allele frequencies for various subset of samples. In particular, allele frequencies have been computed for each of 4 major ethnic groups and for each of 181 countries represented in our simulated data set.

Masking genomic regions with excess of rare variants Disease-causing variants are not uniformly distributed across the genome [5]. They are often clustered in certain parts of the gene, such as selected exons or protein domains, as those functional element of the genome are more likely to be protein-coding. These regions are usually characterized by a depletion of rare variants in control databases [25, 26]. Analogously, an excess of rare variants indicates tolerant, less critical regions in which ~~we~~ one should not expect disease-causing mutations. Filtering variants located within these commonly mutated regions reduces the number candidates and therefore can improve the final interpretation [27, 28].

The focus of the benchmarking was the ability of the data warehouse to compute the cumulative frequencies of rare, predicted deleterious variants in different types of genomic regions such as exons, genes and cytogenetic bands. This information can be further utilized to determine genomic intervals of higher then expected mutational rate [29]. It is worth noting that the same calculations can be further repeated for any subset of samples, e.g. individuals from selected ethnic groups or countries.

Association tests Multiple statistical methods have been developed to support novel disease gene discoveries in large case-control analysis of sequencing data [10, 11, 12, 13, 14]. Classical GWAS approaches aim at identifying single variants, for which allele frequency differ significantly between cases and controls. The main drawback of these methods is a lack of power while dealing with very rare variants [30]. To overcome this issue, various aggregation tests have been proposed. They analyze the cumulative effects of multiple variants in a gene or a genomic region, either by collapsing information from multiple variants into single score (Burden test [10, 11, 12]) or evaluating aggregated score test statistics of individual variants, such as C-alpha[13] or SKAT[14]).

An important issue for aggregated tests is selecting an appropriate subset of variants to be tested for association [30]. The allele frequency cutoff is usually determined using the information on disease prevalence and expected inheritance model. In order to refine further the subset of variants one can use prediction scores to select the most damaging mutations. However, despite many existing prediction algorithms and a variety of filtering strategies there is no single solution that fits to all studies.

Some types of queries have been run to assess the efficiency of our variant warehouse in performing ~~customized~~ customised region-based association tests. In particular, the search was done for genes or exons enriched for rare deleterious variants in selected disease

populations.

Investigating the depth of coverage Coverage statistics obtained from a large population of samples can be used in many ways, including prediction of Copy Number Variants (CNVs) [31, 32] and detection of regions of poor or variable coverage. CNVs are of great importance in clinical investigation because they often allow to explain patient's phenotype. On the other hand, an information about poorly covered regions can be used to improve the results of association studies [33].

Interactive variant browsing To achieve the clinical applicability of genomic variant knowledge and for real bench-to-bedside impact of personalised medicine, it is necessary to provide clinicians and medical researchers with user-friendly tools for flexible querying and real-time browsing variant information for a currently investigated patient. Tasks such as phenotypic or ontology-based searches, comparing large knowledge bases with local sequenced biobanks of patients or finding associations between drug response and variants must certainly have the basis in the efficient ad-hoc variant database queries. Having efficient querying for large variant databases with convenient data management interfaces may convince clinicians to use more of the accumulated genomic knowledge in their daily practice and will have in consequence beneficial influence on patients and therapies.

1.3 State-of-the-art techniques for distributed data processing

To construct the benchmarks for genomic variant database it is necessary to systematise the current technologies, formats and software tools used in this area. Those relevant aspects are listed with short descriptions below.

1.3.1 File formats and storage engines

Apache ORC (`https://orc.apache.org`) and **Apache Parquet** are the most advanced type-aware columnar file formats used in the Hadoop Ecosystem stored using Hadoop File System (HDFS). Both exhibit many design similarities such as pluggable data compression mechanism (e.g. Snappy, ~~zlib~~ gzip/Zlib or LZO), data type specific encoders and support for nested data structures. Apache ORC and Parquet are also widely adopted and many distributed query engines provide support for both, including Apache Spark, Apache Hive and Presto. ORC introduces also a lightweight indexing that enables skipping of complete blocks of rows that do not satisfy the query predicates. However, there has been no consensus yet on whether one of them is superior in terms of performance. Recent studies ([34]) suggest that ORC might be significantly faster (ca. 1.3-5.8x). **Apache Kudu** ([35]) is a novel open source storage engine for structured data which supports low-latency random access together with efficient analytical access patterns. It can be run in parallel with the existing HDFS installation.

1.3.2 Query engines

Among the modern query engines the differentiating factors are e.g. query performance, memory requirements, compatibility or application programming interfaces. In order to choose a subset of engines to be tested with the benchmark data the list of requirements has been prepared:

- ANSI SQL or its dialect as a querying language,
- ODBC/JDBC availability provides interoperability with analytical tools like R, visualization and reporting solutions,
- I/O operations with HDFS file system,
- support for both popular columnar storage: Apache ORC and Parquet,
- support for Apache YARN (*Yet Another Resource Negotiator*) to provide easy and efficient resource sharing among different jobs running on a cluster the same time,
- support for Hive metastore to provide an abstraction layer over physical storage model details,
- at least basic data access authorization.

~~The query engines that has~~ Big data query engines can be divided into four main categories. Historically, the first group introduced an implementation of MapReduce paradigm for executing SQL queries and despite the fact that offers in many cases poor performance it is still widely used because of its maturity and stability. DAG-based category that is a natural successor of the legacy MapReduce approach is currently under active development and seems to becoming the most popular nowadays. MPP-like engines that has is its origin in dedicated analytical appliances (e.g. Netezza, Greenplum, Teradata). The last category are OLAP cubes solutions that store precomputed results (aggregates) using distributed storage systems. The query engines that have been initially evaluated as candidates for including in the benchmark are as follows~~.~~:

Hive is a data warehouse software that enables querying and managing large datasets in a distributed storage. It provides a metastore which can keep the information on data specific parameters such as tables schema, file format or location of the files. Hive can be operated with HiveQL, highly similar to ~~ASCII~~ ANSI SQL. There are various execution engines where HiveQL queries can be run, such as MapReduce, Tez or Spark. Among those, MapReduce is the only execution engine that is supported by all Hadoop distributions (i.e. Hortonworks and Claudera) and therefore only this engine was included in our benchmark.

Hive on MapReduce Hive initially had used MapReduce as the execution engine. MR introduced the paradigm [36] of writing distributed algorithms using two phases: map and reduce. Hive transforms each query into multiple stages consisting of both phases. In case of MapReduce each stage is run independently with sub-results persisted which may lead to IO overhead.

~~**Hive on Tez** is intended to solve the problem of frequent I/O access as well as better memory utilization. This is achieved by applying the idea of directed acyclic graphs (DAG) as a key concept of execution architecture. Programs are embodied as a graph where vertices represent the program logic and edges the data movement. Such approach can be combined well with the model of Hive stages.~~

SparkSQL Apache Spark was designed to solve similar problems as Apache Tez and also utilities the concept of DAG's. It is based on the concept of Resilient Distributed Datasets [37]. Spark apart from running directly Hive queries has its own optimizer for HiveQL called Catalyst. It also puts great emphasis on memory usage with project Tungsten that uses off-heap memory. Recent major performance improvement introduced in the Spark 2.x branch called whole stage code generation was a reason for including two Spark releases (branch 1.6.x - still widely used and 2.1.x the most recent one as of writing) in the benchmarking procedure.

Apache Presto is a project that was not aimed to replace MapReduce as such but to improve interactive analytical queries on large datasets. It allows querying data sources of different sizes from traditional RDBMS and distributed storages like HDFS. Presto also aims to be ~~ASCII~~ ANSI SQL compliant, thus it does not support HiveQL. It is a columnar execution engine initially developed at Facebook and supported by Teradata. It can connect to Hive metastore with a connector.

~~**Apache Drill** is another columnar execution shipped by default with MapR Hadoop distribution. It is unique features encompasses dynamic schema discovery (it can be discovered during runtime) as well as runtime query code compilation. It can inter-operate with Hive tables using a dedicated storage plugin.~~

Apache Impala similar to Presto puts a lot more effort on interactive analytics but it has much more limited support for file formats and data sources. Most notably it lacks support for Apache ORC file format. According to benchmark [34] it is however slower than Presto with Apache ORC as storage in terms of wall time.

Apache Kylin is a distributed OLAP cube solution developed upon Hive and HBase software. It provides a web user interface for both logical (dimensional modeling) and physical (noSQL database table storage) design. Cuboids are computed using map-reduce jobs and loaded into key-value store for fast retrieval. Queries that cannot be answered using OLAP cube can be rerouted to Hive for runtime processing.

MonetDB is a parallel, analytical RDBMS with a columnar-oriented data store. Over the years (project was initiated in the 1990s) it has introduced a great number

of unique features e.g. CPU-tuned query execution architecture, usage of CPU-caches, run-time query optimizations just to name a few. An optional SAM/BAM module for processing of sequence alignment data has been also released recently [20].

~~Query engines comparison query engine version ORC/Parquet JDBC/ODBC YARN security Apache Hive 1.2.1 x x x xApache Spark 1.5.2 x x xx Presto 0.128 x x x/- x/- Apache Drill 1.4 x x - x Apache Impala cdh5.3.9 -/x x x x Apache Kylin 1.2 x x x x MonetDB 11.21.11 N/A xN/Ax~~

Table 1: Query engines comparison

query engine	version	ORC	Parquet	Kudu	JDBC/ODBC	YARN	security
Apache Hive	1.1.0	+	+	-	+	+	+
Apache Spark	1.6.3/2.1.0	+	+	+/-	+	+	+
Presto	0.169	+	+	-	+	+/-	+/-
Apache Impala	2.8.0	-	+	+	+	+	+
Apache Kylin	1.2	+	+	-	+	+	+
MonetDB	11.21.11	N/A	N/A	N/A	+	N/A	+

1.3.3 Major limitations and challenges in the modern distributed database systems

Although distributed computing research area has been developing rapidly for the last 2-3 years, still there are many challenges and limitations that designers and developers of the system should to be aware and which need to be addressed in the future shapes of the software:

- cost-based query optimization is still in its infancy when compared to classic RDBMS – there is still very often a need for manual query tuning like table joins reordering or queries reformulation,
- ANSI SQL conformance is often not yet fully satisfied, which leads to situations where one query needs to be customized for each execution engine,
- many analytical/window functions are missing or named differently,
- distributed queries launch overheads – there is still a lot of effort put into providing more interactive user experience as known from classic RDBMS,
- engines self-tuning features are also not yet implemented which very often results in manual, time-consuming triaging and tuning on the level of engines as well as single queries,

- in most of the cases the underlying storage layer is either optimized for fast sequential reads or random access patterns (Apache Kudu is an exception) and thus sometimes data need to be duplicated.

2 METHODS

2.1 Base data model

In the area of data warehouses many design patterns have been proposed [38, 39] that can be applied in the prototype with some adjustments, specific to the requirements of distributed computing model and query engines. The main issue to be solved are slow joins, which should be preferably replaced with filtering or map-joins. One of the solutions it is to apply the star schema which can lead to executing map-joins when the dimension table can fit into the memory. Figure 1 depicts the star schema of the prototype. Dimension tables are also designed to enable implementation of hierarchies for flexible adjusting of an area of interest, e.g. for the geography dimension one can query over region → subregion → country or for genomic position gene → transcript → exon → chromosome → position.

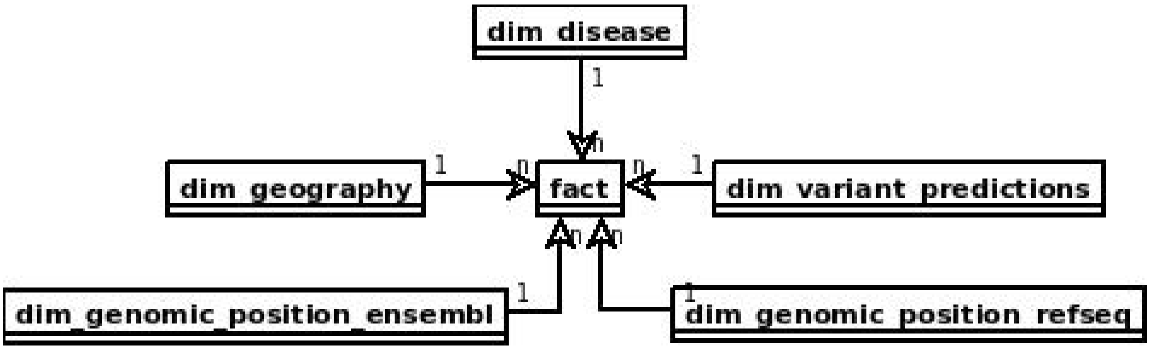

Figure 1: Proposed star schema of the genomic variant data warehouse, with central fact table and tables modelling patients' genotypes and phenotypes and genomic variant annotation to RefSeq and Ensembl.

The *fact* table contains information retrieved from VCF files, such as chromosome, position, reference and alternative alleles, depth of coverage, genotype and genotype likelihoods. It also includes references to all the dimension tables.

The table *dim_geography* represents world region of the patient divided into hierarchical areas. That type of patient information may be relevant in case of population genomics studies From clinical perspective it may help to identify population specific polymorphisms and trace the origin of causative variants, e.g. in epidemiology.

Both *dim_genomic_position_ensembl* and *dim_genomic_position_refseq* cover referential genes and transcript annotations as available in RefSeq an Ensembl databases, respectively. Each record corresponds to a single exon and contains information on its genomic

location and associated transcript's data. Exons included in canonical transcripts are indicated by "iscanonical" flag. The transcripts with "ismerged" flag, are results of overlapping all transcripts that map to the same HGNC gene symbol.

Table *dim_disease* represents a set of OMIM diseases. This table models all the phenotypic and disease information about the patient and sample that can be possibly stored in a genomic data warehouse. This may be extended into a set of data tables, including phenotype ontology and all the clinical parameters relevant for the diseases of patients whose samples are stored. This is the most obvious direction of development of the warehouse structure for practical applications.

The *dim_variant_predictions* contains variant information that is available in dbNSFP database [40] with selected results from some of the major predictors available.

The full definitions of all the tables in the database schema can be found in the project results repository (see Supplementary Information for details).

2.2 Data model optimizations

Base data model organized as a classic star schema is mainly suitable for running queries that require the highest granularity of data but using only a small subset of rows from the fact table. Queries that perform full-table scans over fact table in order to calculate aggregated measures over e.g. geographical items or genomic regions can benefit from being rewritten to be run over aggregation tables. It can be further optimized by introducing aggregation tables that have been pre-joined with some of the most often used dimensions. This can be particularly beneficial in the case of high or ultra-high cardinality dimensions like *dim_genomic_position* or *dim_variant_prediction*. Last but not least, the base data can be transformed into OLAP cubes storing all aggregates along predefined query patterns for running fast *slice and dice* operations. To address all the needs above, four levels of data storage have been introduced:

1. raw data of genotype calls (raw) – is a raw, not aggregated fact table with the highest granularity and references to all the dimension tables,
2. aggregation tables level (aggr) – storing all ~~e.g.~~ variant counts aggregated by ~~country~~ countries, exons, diseases, and keeping references to all dimension tables,
3. aggregation and full denormalization level (aggr+denorm) – the aggregated fact table with pre-joined dimension tables stored as one table,
4. OLAP cube with all aggregates pre-computed and stored in noSQL database (Kylin).

2.3 Construction of benchmarks

2.3.1 Cluster infrastructure overview

Hardware All the test have been run using a 6 node cluster (5 data/processing nodes and 1 used as master and name node). Machines were equipped with 2xE5-2650 CPU resulting in 16 cores/32 threads and 256GB of RAM. Each node had local 6 hard drives in RAID0 (400GB of disk space) mode with peak throughput around 1.3GB/s of sequential read speed. Network interconnect allowed stable transfer at around 200MB/s.

Software ~~Hortonworks Data Platform (HDP) 2.3~~Cloudera (CDH) 5.8.2 distribution were installed ~~using custom Docker images based on Centos 6.6 with Hadoop 2.7.1, Hive 1.2.1~~with Hadoop 2.6.0, Hive 1.1.0, HBase 1.1.1, Kudu ~~0.7.0~~ 1.0.1 and Zookeeper 3.4~~.6~~ .5 as main software components. ~~Apache Spark version were upgraded to 1.5.2 (instead of 1.4.1 shipped with HDP). Docker 1.8.3 was used together with flannel () 0.5~~Versions of other components are summarized in Table 1. Please, note that in the text Spark in version 1.6.3 ~~for providing virtual network between containers running on different physical nodes.~~ is referred to as Spark 1 and Spark in version 2.1.0 as Spark 2.

2.3.2 Query engines

~~Three of general purpose query engines (~~

The benchmark measures the performance of four distributed query engines described above, i.e. Apache Hive (~~on Tez~~MapReduce), Apache Spark ~~, Presto) described above have been used in all the tests. Apache Impalawas used to provide an access to Apache Kudu storage engine. Apache Hive on MR has not been included because it is generally slower than Hive on Tez and not suitable for running interactive analytical queries.~~ (versions 1.x and 2.x), Presto, and Apache Impala. First three engines were tested using two different file formats: ORC and Parquet. Apache Impala, which does not support ORC, was tested in configuration with Parquet and Apache Kudu.

In addition, in the case of queries against aggregation tables, MonetDB database was used as a a baseline to compare performance of distributed query engines versus one of the fastest parallel, columnar but still single-node relational databases. Distributed cube OLAP solution – Apache Kylin has been also reviewed to indicate a possibility of reducing query times even more, with the aim of execution time below a single second.

2.3.3 Data warehouse physical data model details

In all the tests queried tables were stored in either ORC or Parquet format with gzip compression and registered as Hive tables in Hive Metastore.

Table 2 presents the details of the physical tables stored in Hive and MonetDB.

~~Physical data model properties. Sizes in GB. TablerowscolumnsORC-zlib size PQ-zlib~~

size Kudu-zlib sizeMonetDB size fact 4827005561 14 45.7 36.6 76.3 69.6* fact_agg_counts 230380448 18 3.1 3.2 8.7 25.7 fact_agg_counts_dims 230380448 67 10.9 10.1 34.8 72.1 dim_gen_pos_ens 1519361 12 0.0097 0.0122 0.366 0.064 dim_gen_pos_rs 725811 12 0.0064 0.007 - 0.041 dim_geography 249 7 0.000004 0.000004 0.000012 0.007 dim_disease 7569 3 0.000104 0.000113 0.000348 0.003 dim_variant_predict 369006734 28 10.8 11.8 19.1 54.2

Table 2: Physical data model properties. Sizes in GB.

Table	rows	columns	ORC-Zlib size	PQ-Zlib size	Kudu-Zlib size	MonetDB size
fact	4827005513	14	24.9	21.7	76.3	69.6*
fact_agg_counts	230380448	20	3.1	3.2	8.7	25.7
fact_agg_counts_dims	230380448	67	10.9	10.1	34.8	72.1
dim_gen_pos_ens	1519361	12	0.0097	0.0122	0.366	0.064
dim_gen_pos_rs	725811	12	0.0064	0.007	-	0.041
dim_geography	249	7	0.000004	0.000004	0.000012	0.007
dim_disease	7569	3	0.000104	0.000113	0.000348	0.003
dim_variant_predict	391271826	28	10.8	11.8	19.1	54.2

In the case of MonetDB only $1 * 10^9$ rows (approx. 1/5 of the dataset) have been loaded to the fact table, while the rest of the tables (aggregation and dimension) were the exact copies of those stored in Hive. It was because of the disk space constraints, as MonetDB does not provide any data compression mechanism. It has been estimated that the total size of fact and dimension tables would exceed 400GB that was attached as local storage.

The first level (*fact*) table enables running most general queries even for single samples. The next two levels (2-3) (*fact_agg_counts* and *fact_agg_counts_dims* are pre-aggregated by all dimension's foreign keys. Levels 1-3 can be queried using different computing engines (Hive on TezMapReduce, SparkSQL, Presto) and the data is stored as one of columnar storage such as Apache ORC or Parquet, whereas the 4th level is implemented using Apache Kylin which with HBase as a storage.

The size of the Kylin OLAP cube was 47.6GB and it took approximately 6.5h to build it. In case of Apache Kudu there was a need to add an artificial primary key in case of aggregated tables (level 2,3).

2.3.4 Formulation of queries and testing

The performance of the variant, data warehouse has been tested using 5 12 types of queries that correspond to biomedical issues discussed in the previous section. The detailed description of queries and engine-specific versions of SQLs are available in the results repository.

- **Q1: Allele frequencies – breakdown by geographical region.** For every variant the set of population specific allele frequencies corresponding to 4 continents (subquery A) or 181 countries (subquery B) is calculated. This type of data

can be used to identify and flag common polymorphisms observed only in selected populations.

- **Q2: Cumulative frequencies per genomic interval.** The number of distinct rare (allele frequency $< 1\%$), deleterious (predicted as damaging by FATHMM) variants and their cumulative allele frequencies are computed for every single transcript (subquery A) or exon (subquery B) in the human genome. This information may help to detect the commonly mutated regions that could be masked in the clinical investigation of patients variant data.

- **Q3: Enrichment of variants in disease population.** The aggregated variant counts are computed for selected subpopulation of disease patients for every transcript (subquery A) or exon (subquery B) in the human genome. These queries provide substrates for aggregation tests and can be easily used to identify genes or exons with excess of damaging variants in disease population.

- **Q4: Distribution of variant's coverage across genomic intervals.** Minimum, 25-th percentile, median, 75-th percentile and maximum depth of coverage across all samples is computed for every transcript (subquery A) or exon (subquery B) in the human genome. These queries, allow to locate poorly or variably covered regions that may be a cause of inflation of false positive values in association studies.

- **~~Q5~~Q5-Q12: ~~Browsing individual's variants~~Set of queries on the fact table.** ~~The~~ In addition to the 8 complex queries (4 query families) described above, the benchmark includes a set of 8 queries that act on the fact table only, i.e. without joins with dimension tables. In particular, **Q5** returns the list of all variants from the single sample and the given genomic region~~are extracted~~. This simulates a typical scenario in which clinician/analyst explore patient's variants in the gene/region of interest. It is important that such a simple genome range queries are processed efficiently, possibly providing an answer in less than a few seconds. Remaining queries can be useful in other types of exploratory analysis or in a process of quality control. These queries return:

  - **Q6**: the number of variants' occurrences corresponding to the same substitution type (e.g. C > G) in all samples,
  - **Q7**: the number of distinct variants per chromosome observed in all samples,
  - **Q8**: the number of variants for which a ratio of variant to total reads exceeds 90 % in the given sample,
  - **Q9**: the ratio of heterozygous to homozygous variants on X chromosome in the given sample,

- **Q10**: the number of variants per sample for a given chromosome,
- **Q11**: the number of different genes containing variants per sample,
- **Q12**: the number of variants per chromosome for a given sample.

2.3.5 Test set properties and its generation

The largest publicly available variant datasets (such as ExAC) contain data from more than 50,000 of samples. Unfortunately, ExAC does not provide sample level genotype data. Downloadable VCFs contain variant allele frequencies across different populations, however no genotype data for individual samples are reported. To generate dataset that has similar properties to the ExAC one, a data simulator has been implemented that uses real population-specific allele frequencies extracted from ExAC.

For the purpose of testing, an artificial SNV data set has been generated, simulating 50,000 whole exome sequences. To ensure the actual distribution of genomic variants in geographical populations, the ethnic-specific allele frequencies available in ExAC database have been used. This simulation procedure consists of 3 steps:

First, every sample is assigned to one of four ethnic groups, i.e. Europeans, Americans, Asians or Africans. Then, samples within an ethnic group are associated with countries, which are randomly selected with respect to relative population sizes.

Subsequently, variants are simulated based on information present in the dbNSFP [40] including chromosomal position, reference/alternative alleles and allele frequencies from ExAC. For every variant in dbNSFP the genotype has been selected with a probability $p(af)$ and corresponding to the proper ethnic group allele frequency:

$$p(af) = \begin{cases} 1 - 2 \cdot af + \cdot af^2 & \text{for genotype } 0/0 \\ 2 \cdot af \cdot (1 - af) & \text{for genotype } 0/1 \\ af^2 & \text{for genotype } 1/1 \end{cases}$$

For every genotype generated in previous step the total depth and allelic depth of coverage have been simulated. For a given position of the variant~~we fixed~~ , the average value of the total number of reads has been fixed, using information reported in ExAC about a mean depth of coverage at this location.

2.3.6 Test procedure automation

SQL-dialect specific version of all the queries have been run using all query engines and in case of aggregated (storage level 2), aggregated and denormalized (storage level 3) also using MonetDB. Queries on storage level 3 have been also tested using the Kylin cube.

To execute queries using selected engines and storage formats there has been prepared a parametrized YAML-based configuration file for each query (see `https://github.com/`

`ZSI-Bio/variantsdwh` for details). This enabled us to automate the process of testing different combinations of query engines and storage formats. Proposed automation framework consists of Scala utility that reads parametrized SQL query in a YAML file (together with additional metadata information) and executes it using a selected execution engine (using a JDBC interface) against tables stored in a desired file format a specified number of times. In order to even further automate the benchmarking process additional shell scripts were developed that execute end to end scenario, e.g. start Spark 1 Thrift server, run queries, stop it, and repeat the same steps with Spark 2. Framework is also shipped with tools that can generate the physical model, populate dimension tables and generate fact table of a given size. Data analyses and visualisation step is implemented as a set of R scripts that process CSV output of the benchmark procedures.

In the case of Hive tables stored as ORC or Parquet format gzip/Zlib compression has been used in both cases. Each query from the test set has been run several times and average value has been calculated. Disk buffers at operating system level were purged before each query launch.

3 RESULTS AND DISCUSSION

After performing the queries with the selection of database and query engines, a number of conclusions and recommendations can be formulated. The numeric results of the benchmarking tests can be found in the table

3.1 File formats and storage engines

Testing both ORC and Parquet file formats revealed that there exist serious differences in their implementations between the computing engines. The same query run using a different file format can slow down even by factor of approximately ~~4x (Q1), but usually the difference is ca.~~ 1.5-2x (see Table 3). It can be observed that Apache Hive performs better using ORC than Parquet~~for larger tables whereas poorer for smaller aggregation tables~~. Apache Spark ~~almost~~ always favours Parquet format, so does Presto with ORC but here the difference seems to be less obvious (with the exception of queries without join operations where the difference can be significant). The difference in size of the compressed files (using gzip/Zlib algorithm) were comparable, varied depending on a table but in the case of the fact table reached the maximum value of ca. 20%. ~~In general, using Parquet over ORC file format is recommended for running analytical queries. When both sequential reads and random access patterns are of interest, then Apache Kudu storage engine seems to be the best option available. It outperforms ORC and Parquet formats by an order of magnitude in case of genome range queries (Q5) leveraging a primary~~

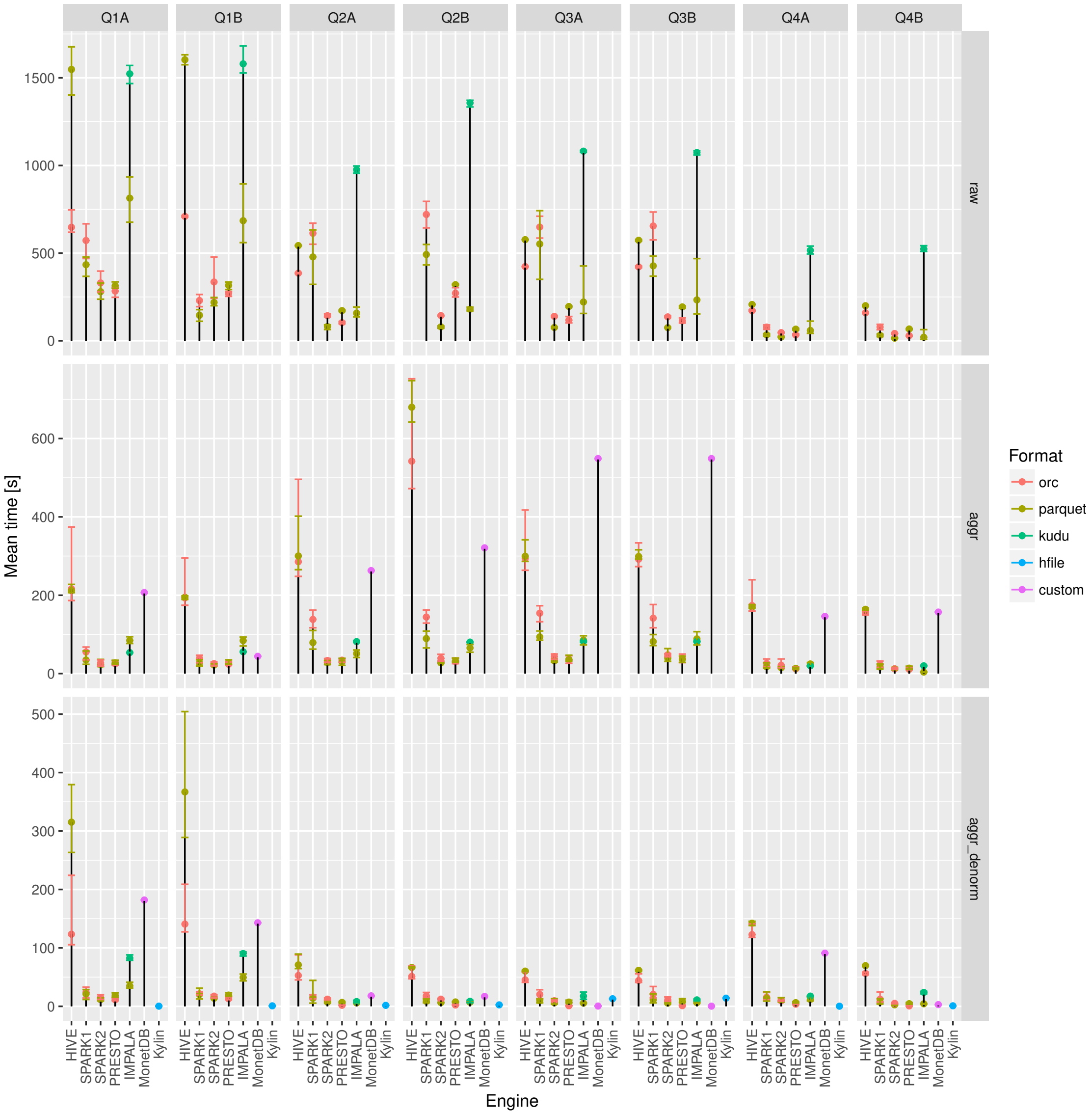

Figure 2: ~~Relative performance (execution time) of~~ Execution times for all the query engines and file formats for the queries Q1-Q4 over raw, aggregation and denormalized tables with MonetDB as a baseline. For a given configuration (query engine and file format) each query was executed 3 to 5 times. Different colors were used to show the average execution times for different file formats. In addition, lower and upper bounds of error bars indicate the minimum and maximum query execution time, respectively.

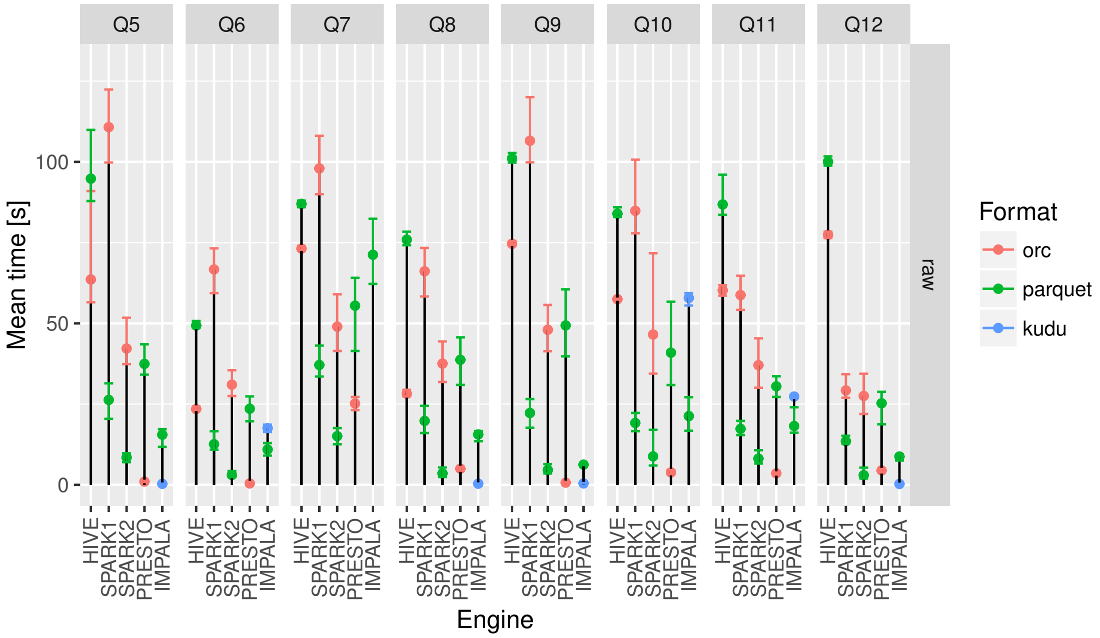

Figure 3: ~~Panel A,B: Distribution of execution~~ Execution times for all the query engines and file formats ~~(~~for queries ~~Q1-Q4~~Q5-Q12. For a given configuration (query engine and file format) ~~; Panel C: Execution~~ each query was executed between 3 to 5 times. Different colors were used to show the average execution times for different file formats. In addition, lower and upper bounds of error bars indicate the minimum and maximum query ~~Q5 (variant browsing)~~ execution time, respectively.

~~key. Of note, Apache Kudu does not use HDFS for data storage and currently it is fully supported only by Impala query engine.~~

In summary, Parquet-based file formats for storing genomic information (as e.g. in ADAM) are a good choice for running ~~analytical queries~~ complex analytical queries (e.g. Q1-Q4) whereas are not really suitable for fast random access patterns, e.g. interactive variants browsing for a given sample identifier and genomic position ranges (Q5). When choosing this file format Apache Spark 2 seems to best query engine that in most of the cases outperforms both Apache Spark 1 and Apache Impala. The other option that is currently worth considering is combination of ORC file format with Presto query engine. In most of the cases it is slower in case of the queries on raw data requiring joins with dimension tables but on the other hand is more performant in single table scan operations and data browsing. Neither of these combinations of query engine/file format can compete with Apache Impala/Kudu configuration in terms of fast data browsing that can offer sub-second responses in most of the cases.

3.2 Query engines

None of the evaluated computing engines was an obvious winner in all the queries and storage levels (see ~~Figure ?? andFigure ??~~Figures 2 and 3 and Table 3). The results can

be summarized as follows:

- Presto is ~~more suitable~~ a perfect choice for simpler queries (with fewer or no joins) or smaller tables (e.g. aggregated and denormalized), on the other hand it is ~~by no means~~ also suitable for complex queries with many joins ~~, as it failed or timed out when running test queries on highest granularity fact table~~but is slower than Apache Spark 2,

- Apache ~~Tez~~ Hive (MapReduce) is particularly good at complex queries with joins run on large fact tables ~~, only Apache Spark could compete with it in a few cases. Still it~~ but still slower than other DAG-based and MPP-like solutions. It does not excel at interactive, simpler queries when it always was slower and the difference even more visible,

- Apache Spark ~~showed the greatest variability in execution times but it~~ 2 seems to be most general purpose tool in the study. It is suitable for both heavy processing ~~(although in many cases slower than Apache Tez)~~ and comparable to Presto when interactivity is of importance. A clear performace boost when compared to Apache Spark 1 was observed - in almost all the test cases it was faster and in some the difference was even 5-6x,

- Apache Kylin whereas is not as flexible as fully-fledged query engines with properly designed OLAP cube can be truly interactive tool with sub-second response times,

- all of the query engines show superior performance over MonetDB in case of running star-queries over the aggregation tables with an average speedup approx. 3-7x, in case of queries against aggregated and denormalized tables execution times converged, in a few queries MonetDB proved to be equally fast as Kylin. MonetDB seems to exceptionally well deal with queries run against a single table.

- Apache Impala was the only query engine that was used to access data stored in Apache Kudu. ~~It showed one of the lowest variation of execution times (in case both Parquet and Kudu) while still being comparable to other engines in case of analytical queries (Q1-Q4).~~ When used together with Apache Kudu, it was the ~~only~~ best combination capable of answering genome range queries (Q5) in a truly interactive way (<1s). It also offered comparable execution times to the Apache Spark 2 and Presto in case of single table queries in many cases.

~~Queries execution times for Apache Hive, Apache Spark, Presto, Kudu, MonetDB and Apache Kylin Level MonetDB sKylin sFormat ORC Parquet ORC Parquet ORC Parquet Parquet Kudu Custom HFile raw **376** 1537 2400 >1h 624 576 1093 678 – aggr 63 58 59 44 21 **13** 47 90 207 – aggr+denorm 63 54 41 44 21 13 52 90 182 **0.32**raw 732 894~~

Table 3: Queries average execution times for Apache Hive, Apache Spark, Presto, Impala, MonetDB and Apache Kylin

Query	Level	Hive on MR [s]		Presto[s]		Spark 1[s]		Spark 2[s]		Impala [s]		MonetDB [s]	Kylin [s]
	Format	ORC	Parquet	ORC	Parquet	ORC	Parquet	ORC	Parquet	Parquet	Kudu	Custom	HFile
$Q1_A$	raw	648	1548	283	314	572	434	330	**280**	814	1523	–	–
	aggr	216	212	25	28	55	35	27	**23**	85	54	207	–
	aggr+denorm	123	315	11	19	24	21	15	11	35	83	182	**0.32**
$Q1_B$	raw	710	1603	270	316	230	**145**	335	219	685	1580	–	–
	aggr	193	195	24	28	39	27	25	**22**	84	56	44	–
	aggr+denorm	141	367	13	19	21	20	17	14	49	90	143	**0.85**
$Q2_A$	raw	386	543	103	172	613	478	143	**81**	157	976	–	–
	aggr	285	300	34	29	138	79	33	**28**	51	82	263	–
	aggr+denorm	53	71	1.81	6.74	16	14	12	7.16	5.82	8.2	18	**1.7**
$Q2_B$	raw	failed	failed	271	320	721	492	144	**79**	180	1357	–	–
	aggr	542	680	31	33	144	89	39	**27**	65	80	321	–
	aggr+denorm	51	67	**2.24**	7.49	18	8.59	12	6.37	6.91	8.64	17	2.5
$Q3_A$	raw	423	577	118	196	649	552	139	**76**	221	1082	–	–
	aggr	294	300	35	36	154	94	44	**32**	85	82	549	–
	aggr+denorm	45	60	1.01	7.25	20	9.62	9.67	5.94	5.43	18	**0.5**	13
$Q3_B$	raw	422	573	113	193	655	427	136	**75**	233	1074	–	–
	aggr	292	299	39	**36**	141	82	48	39	89	82	549	–
	aggr+denorm	44	62	1.18	8.63	21	11	11	5.47	6.3	11	**0.3**	14
$Q4_A$	raw	171	207	33	66	78	34	47	**21**	59	516	–	–
	aggr	174	170	**12**	13	26	17	21	15	25	20	146	–
	aggr+denorm	123	142	3.66	6.36	16	14	9.51	9.72	12	18	91	**0.26**
$Q4_B$	raw	160	200	29	67	79	32	42	**15**	20	524	–	–
	aggr	155	164	11	14	23	16	12	12	**3.81**	20	157	–
	aggr+denorm	56	70	**0.71**	4.58	12	7.19	5.1	2.6	4.35	24	3	0.91
Q5	raw	64	95	1.01	37	111	26	42	8.49	16	**0.33**	–	–
Q6	raw	23	49	**0.45**	24	67	13	31	3.05	11	18	–	–
Q7	raw	73	87	25	55	98	37	49	**15**	71	308	–	–
Q8	raw	28	76	5	39	66	20	38	3.55	16	**0.36**	–	–
Q9	raw	75	101	0.69	49	107	22	48	4.61	6.26	**0.46**	–	–
Q10	raw	58	84	**3.86**	41	85	19	47	8.82	21	58	–	–
Q11	raw	60	87	**3.58**	30	59	17	37	8.13	18	27	–	–
Q12	raw	77	100	4.47	25	29	14	28	3	8.78	**0.27**	–	–

~~>1h >1h 236 **213** 895 505 – –aggr 80 57 81 66 18 **13** 50 92 44 –aggr+denorm 64 61 43 43 21 14 58 78 143 **0.85**raw 201 332 >1h >1h 548 473 **187** 532 – –aggr 119 122 59 **36** 250 261 44 78 263 –aggr+denorm 47 36 10 8 15 9 6 20 18 **1.7**raw **237** 459 >1h >1h 840 720 247 798 – –aggr 139 142 157 150 292 251 **58** 100 321 –aggr+denorm 58 35 12 9 15 9 6 19 17 **2.5**raw 217 359 >1h >1h 509 446 **216** 600 – –aggr 134 157 118 146 261 234 **73** 113 549 –aggr+denorm 34 21 62 19 14 9 4 18 **0.5** 13raw 216 359 >1h >1h 900 720 **215** 603 – –aggr 140 173 120 113 264 225 **74** 115 549 –aggr+denorm 49 33 15 9 17 8 4 18 **0.3** 14raw 110 157 132 122 80 **44** 111 305 – –aggr 69 70 69 47 **20** 21 26 37 146 –aggr+denorm 100 61 42 43 20 17 23 26 91 **0.26**raw 96 115 81 79 81 **39** 110 400 – –aggr 52 53 43 **12** 18 14 26 36 157 –aggr+denorm 33 28 7 6 26 8 3 29 3 **0.91**Q5 raw 950 1244 7 35 103 88 19 **0.9** – –~~

3.3 Data compression

The impact of data compression on table size and query execution times has been tested for two best performing configuration, i.e. Presto with ORC and Spark 2 with Parquet.

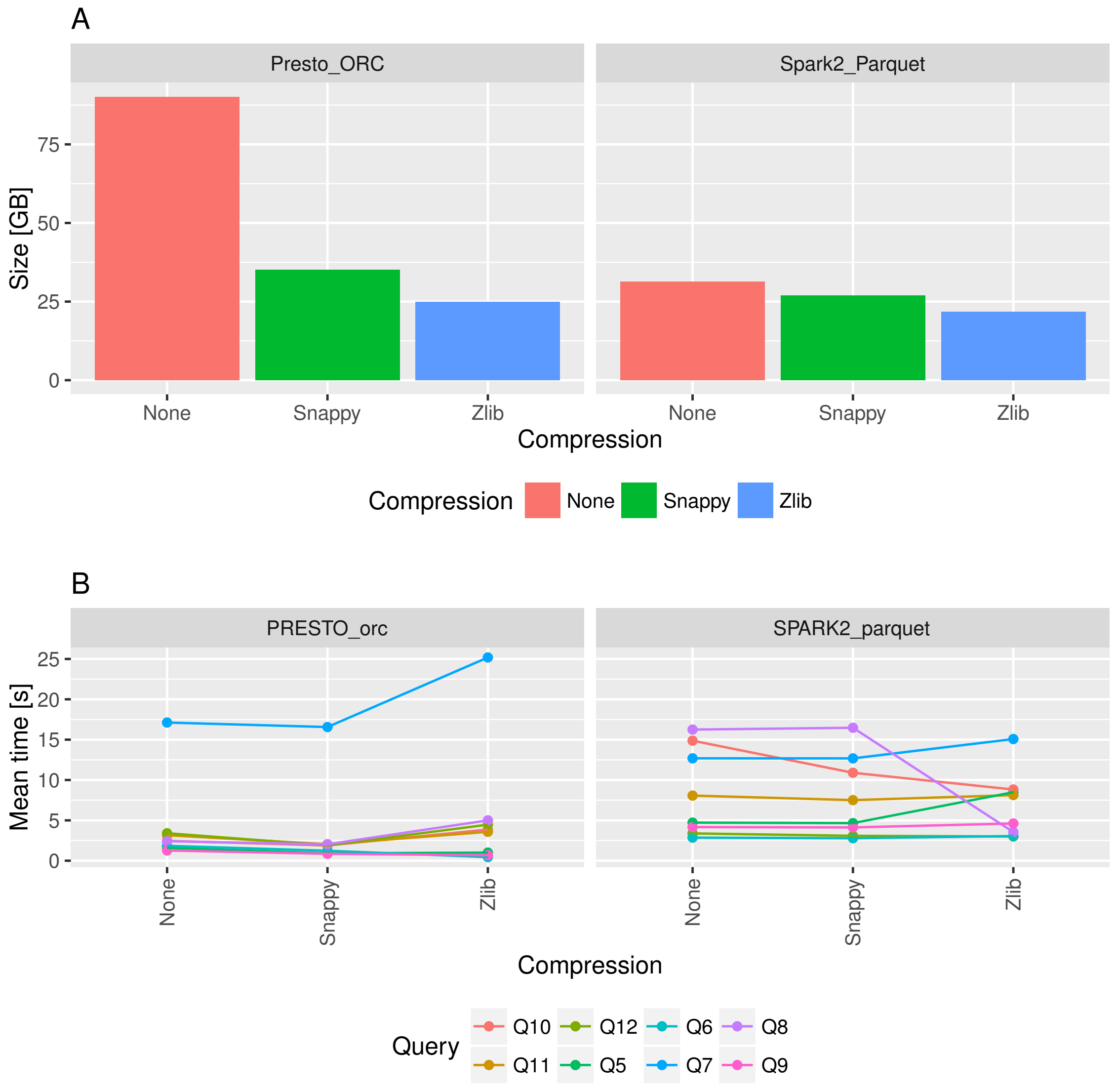

Figure 4: Impact of compression on fact table size and execution times for queries Q5-Q12.

3.4 ~~Data compression~~

Using columnar storage together with gzip/Zlib or Snappy compression can significantly reduce storage requirements (Figure 4A). In comparison to MonetDB the storage space required can be even 5-7x smaller ~~. The negative impact of~~ (Table 2). In general data encoding and compression in Parquet is 15% better than ORC in case of Snappy and gzip/Zlib compression methods. Besides non-compressed data encoded with ORC format can be even three times bigger than non-compressed data encoded with Parquet (Figure 4A).

Comparison of execution times revealed that there are no visible overhead of Snappy data compression on the ~~query performancecan be neglected (data not shown)but it can result in 2-3x storage reduction in case of Parquet file format while compared to the uncompressed one~~performance. On the other hand, gzip/Zlib compression may have either positive or negative impact on query execution times, depending on the query (Figure 4B). In case of queries that require full table scans negative impact of gzip/Zlib compression can be observed, e.g. compare execution times for Q7.

3.4 Data model optimizations

The use of aggregation tables can result in huge speedup when running queries that require full-table scan to compute aggregated measures (Figure 5). In the conducted tests it ranged from 6x in case of ~~Tez to 44x~~ Hive up to 100x in case of ~~Apache Spark with Parquet~~ Presto with ORC file format.

3.5 Application of OLAP cube

Apache Kylin can offer unbeatable performance at a cost of flexibility when running queries following predefined patterns (i.e. hierarchies, groupings, measures). In most of the cases it proved to be approx. 5-20x faster than Presto or SparkSQL. It can be seen as a 'coprocessor' boost component that can offload SparkSQL or Presto by handling predefined but parametrized queries.

3.6 Recommendations for genomic data warehouse designers

Designing a scalable performant analytical system that can handle rapidly growing amount of genomic data is by no means an easy task. In this manuscript a few crucial findings that may be treated as design guidelines have been highlighted:

- There are many competing big data ready file formats, query engines and their combinations can substantially differ in performance characteristics. Moreover, serious performance differences can be observed after upgrade from one version

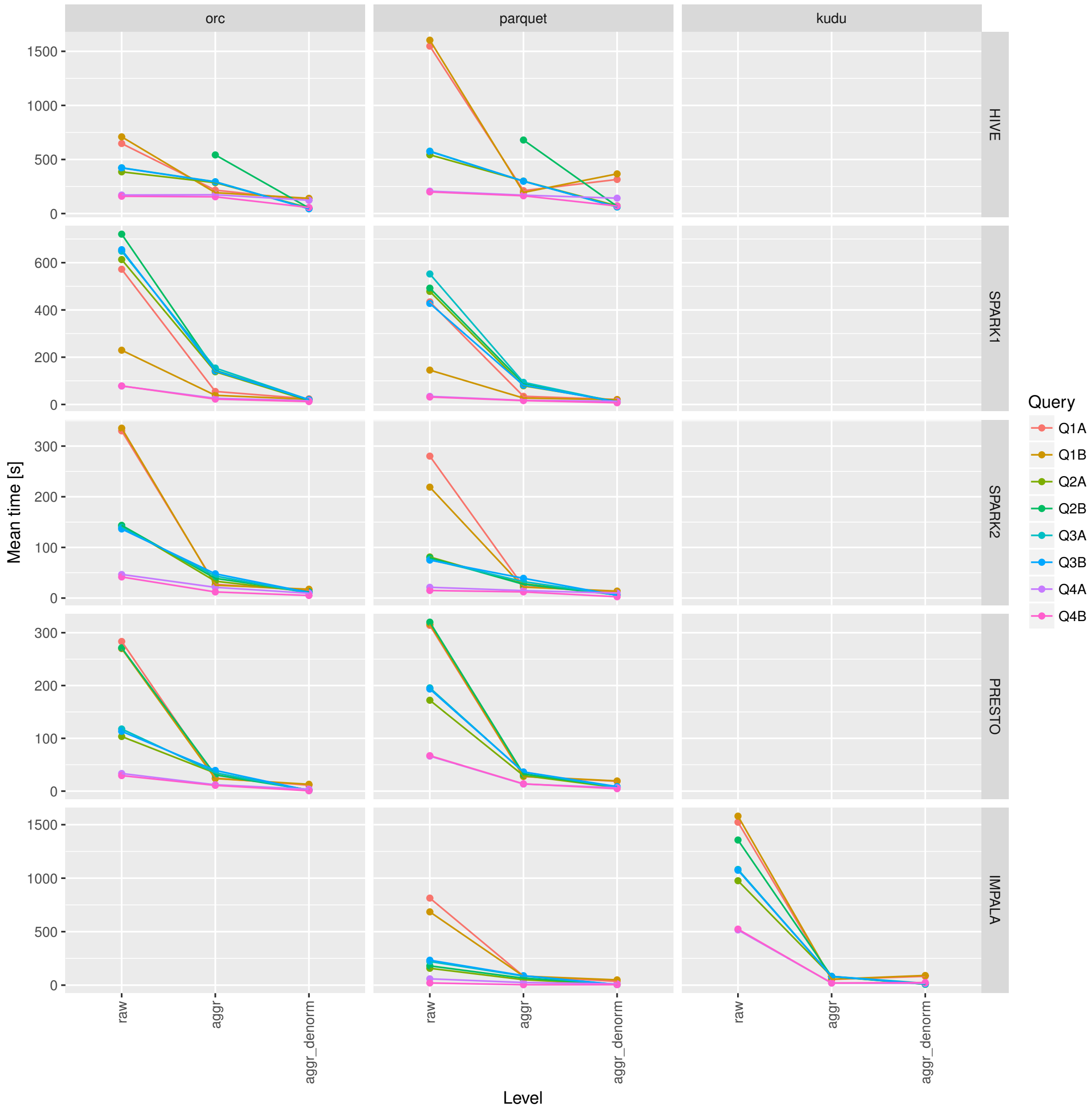

Figure 5: Impact of aggregation and denormalization on query performace for queries Q1-Q4.

of a tool to another. Furthermore, since all of the discussed solutions are truly distributed, also infrastructure characteristics such as network interfaces and storage systems throughput can impact the system performance. This is why it is advisable to use benchmarking frameworks prior taking a final decision on the architecture of the genomic data warehouse.

- Another clear finding is that there is no superior combination of query engine and storage format. Besides, in case of data warehouses solution there is a need for at least three kinds of processing: (i) ETL/ELT (extract, transform, load) processes for loading/refreshing tables, materialized views (aggregation tables), (ii) running large scale analytics, and finally (iii) random records browsing. They all differ in query latency requirements. In case of the first two scenarios, it is acceptable that processing may take longer than a few seconds (e.g. up to few minutes), whereas range queries that are used to populate views of end user interfaces are expected to execute in a fraction of second. Taking into consideration presented results we recommend to use ORC file format together Presto as a tool for running interactive queries and Apache Spark 2 for implementing ETL processes and background queries for answering population scale research questions.

- Distributed machine learning libraries that integrate seamlessly with Apache Spark such as MLlib (`http://spark.apache.org/mllib/`) or Sparkling Water (`https://www.h2o.ai/download/sparkling-water/`) can be recommended for running more sophisticated exploratory analyses.

- Distributed OLAP cubes solution together with denormalized aggregation tables can serve as acceleration layer suitable for plugging into performant end user interfaces.

- In most of the cases usage of data compression (such as Snappy) is advisable as it substantially reduce storage requirements and do not negatively impact the performance. Since the data are stored in a columnar fashion sorting data by low cardinality columns such as chromosome, reference allele, alternative allele together with a sample identifier can improve the compression ratios even further.

- All the tested query engines support JDBC/ODBC standards and with some additional effort can execute the same SQL queries. This provides a possibility of relatively easy way of switching between execution engines.

3.7 Summary and future directions

This study is intended to point out directions for database designers and bioinformaticians wishing to work on genomic big data currently being produced by sequencing. The com-

putational experiment presented in the paper is an initial proof of the utility of modern columnar databases and query engines to genomic variant data warehouses. At the same time, it ~~was pointed out~~ has been pointed out in the experiments results that for specific purposes the data structures and queries can be optimized and various query engines are complementary. The development of new distributed systems is an ongoing process, so ~~such~~ benchmarks as presented in the paper should be run in the future also for the novel solutions that almost certainly will be developed in the software ecosystems. ~~This study is intended to point out directions for database designers and bioinformaticians expecting to operate on genomic big data that currently are being produced by sequencing~~Such a benchmark can be easily updated, since the source code for our automated benchmarking framework along with data simulator, complete testing data set, SQL queries and raw results described above are publicaly available at `https://github.com/ZSI-Bio/variantsdwh`.

~~While~~ It needs to be also clearly stated that there is still a room for improvement in terms of the performance of genomic data warehouse solutions. Big data technologies such as Apache Kudu or more recent one - Apache CarbonData (`http://carbondata.incubator.apache.org`) indicates that it might be possible soon to have one storage format that supports OLAP style queries, sequential scans and random access efficiently. Moreover, both technologies allow performant random updates and inserts of data which would be desirable in many cases. Further improvements in vectorized query processing together with better support for Single Instruction Multiple Data (SIMD) extension available in modern CPUs would result in better performance of query engines. Integration of computation engines with hardware accelerators, such as graphic cards (General-purpose computing on graphics processing units, GPGPU), could be beneficial, especially in case of machine learning analyses.

Since the genomic variants datasets are ~~large indeed , thus~~ indeed large, the execution time optimization can play significant positive role in personalised medicine research and near future applications of large genomic biobanks. ~~It~~ This is not a trivial task, so will require more research and close collaboration between the medical domain experts and creators of modern distributed data processing applications. In particular, knowing the results of this paper, it is highly recommended ~~to do an advance~~ that a definition of query types and templates is created in advance by the working together of database designers with the experts of clinical genomics and ~~test~~ that its performance with particular storage and execution engines ~~.~~ is tested with similar benchmarks.

FUNDING

This work has been supported by the Polish National Science Center grants:
Opus 2014/13/B/NZ2/01248 and Preludium 2014/13/N/ST6/01843 .

References

[1] The ARIC Investigators (1989) The Atherosclerosis Risk in Communities (ARIC) Study: Design and Objectives. *American Journal of Epidemiology*, **129**, 4, 687–702.

[2] Chong, J. *et al.* (2015) The Genetic Basis of Mendelian Phenotypes: Discoveries, Challenges, and Opportunities. *The American Journal of Human Genetics*, **97**, 2, 199–215. doi:10.1016/j.ajhg.2015.06.009.

[3] Kaye, J. *et al.* (2014) Managing clinically significant findings in research: the UK10K example. *European journal of human genetics: EJHG*, **22**, 9, 1100–1104. doi:10.1038/ejhg.2013.290.

[4] Cancer Genome Atlas Research Network *et al.* (2013) The Cancer Genome Atlas Pan-Cancer analysis project. *Nature Genetics*, **45**, 10, 1113–1120. doi:10.1038/ng.2764.

[5] Davydov, E.V. *et al.* (2010) Identifying a high fraction of the human genome to be under selective constraint using GERP++. *PLoS computational biology*, **6**, 12, e1001025. doi:10.1371/journal.pcbi.1001025.

[6] Adzhubei, I. *et al.* (2013) Predicting functional effect of human missense mutations using PolyPhen-2. *Current Protocols in Human Genetics / Editorial Board, Jonathan L. Haines ... [et Al.]*, **Chapter 7**, Unit7.20. doi:10.1002/0471142905.hg0720s76.

[7] Shihab, H.A. *et al.* (2013) Predicting the functional, molecular, and phenotypic consequences of amino acid substitutions using hidden Markov models. *Human Mutation*, **34**, 1, 57–65. doi:10.1002/humu.22225.

[8] Schwarz, J.M. *et al.* (2014) MutationTaster2: mutation prediction for the deep-sequencing age. *Nature Methods*, **11**, 4, 361–362. doi:10.1038/nmeth.2890.

[9] Vaser, R. *et al.* (2016) SIFT missense predictions for genomes. *Nature Protocols*, **11**, 1, 1–9. doi:10.1038/nprot.2015.123.

[10] Morgenthaler, S. and Thilly, W.G. (2007) A strategy to discover genes that carry multi-allelic or mono-allelic risk for common diseases: a cohort allelic sums test (CAST). *Mutation Research*, **615**, 1-2, 28–56. doi:10.1016/j.mrfmmm.2006.09.003.

[11] Li, B. and Leal, S.M. (2008) Methods for detecting associations with rare variants for common diseases: application to analysis of sequence data. *American Journal of Human Genetics*, **83**, 3, 311–321. doi:10.1016/j.ajhg.2008.06.024.

[12] Madsen, B.E. and Browning, S.R. (2009) A groupwise association test for rare mutations using a weighted sum statistic. *PLoS genetics*, **5**, 2, e1000384. doi:10.1371/journal.pgen.1000384.

[13] Neale, B.M. *et al.* (2011) Testing for an Unusual Distribution of Rare Variants. *PLoS Genet*, **7**, 3, e1001322. doi:10.1371/journal.pgen.1001322.

[14] Wu, M. *et al.* (2011) Rare-Variant Association Testing for Sequencing Data with the Sequence Kernel Association Test. *The American Journal of Human Genetics*, **89**, 1, 82–93. doi:10.1016/j.ajhg.2011.05.029.

[15] Schumacher, A., Pireddu, L., Niemenmaa, M., Kallio, A., Korpelainen, E., Zanetti, G. and Heljanko, K. (2014) SeqPig: simple and scalable scripting for large sequencing data sets in Hadoop. *Bioinformatics*, **30**, 1, 119–120.

[16] Wiewiórka, M.S., Messina, A., Pacholewska, A., Maffioletti, S., Gawrysiak, P. and Okoniewski, M.J. (2014) SparkSeq: fast, scalable, cloud-ready tool for the interactive genomic data analysis with nucleotide precision. *Bioinformatics*, p. btu343.

[17] Niemenmaa, M., Kallio, A., Schumacher, A., Klemelä, P., Korpelainen, E. and Heljanko, K. (2012) Hadoop-BAM: directly manipulating next generation sequencing data in the cloud. *Bioinformatics*, **28**, 6, 876–877.

[18] Ameur, A., Bunikis, I., Enroth, S. and Gyllensten, U. (2014) CanvasDB: a local database infrastructure for analysis of targeted-and whole genome re-sequencing projects. *Database*, **2014**, bau098.

[19] Cheng, W.Y., Hakenberg, J., Li, S.D. and Chen, R. (2015) DIVAS: a centralized genetic variant repository representing 150 000 individuals from multiple disease cohorts. *Bioinformatics*, p. btv511.

[20] Cijvat, R., Manegold, S., Kersten, M., Klau, G.W., Schönhuth, A., Marschall, T. and Zhang, Y. (2015) Genome sequence analysis with MonetDB: a case study on Ebola virus diversity. *Datenbanksysteme für Business, Technologie und Web (BTW)*, pp. 143–149.

[21] Dorok, S. (2015) The Relational Way To Dam The Flood Of Genome Data. In *Proceedings of the 2015 ACM SIGMOD on PhD Symposium*. ACM, pp. 9–13.

[22] Massie, M., Nothaft, F., Hartl, C., Kozanitis, C., Schumacher, A., Joseph, A.D. and Patterson, D.A. (2013) Adam: Genomics formats and processing patterns for cloud scale computing. *EECS Department, University of California, Berkeley, Tech. Rep. UCB/EECS-2013-207*.

[23] Dong, C. *et al.* (2015) Comparison and integration of deleteriousness prediction methods for nonsynonymous SNVs in whole exome sequencing studies. *Human Molecular Genetics*, **24**, 8, 2125–2137. doi:10.1093/hmg/ddu733.

[24] Lupski, J.R. *et al.* (2011) Clan genomics and the complex architecture of human disease. *Cell*, **147**, 1, 32–43. doi:10.1016/j.cell.2011.09.008.

[25] MacArthur, D.G. *et al.* (2012) A systematic survey of loss-of-function variants in human protein-coding genes. *Science (New York, N.Y.)*, **335**, 6070, 823–828. doi: 10.1126/science.1215040.

[26] Consortium, E.A. *et al.* (2015) Analysis of protein-coding genetic variation in 60,706 humans. *bioRxiv*, p. 030338. doi:10.1101/030338.

[27] Fajardo, K.V.F. *et al.* (2012) Detecting false positive signals in exome sequencing. *Human Mutation*, **33**, 4, 609–613. doi:10.1002/humu.22033.

[28] Shyr, C. *et al.* (2014) FLAGS, frequently mutated genes in public exomes. *BMC Medical Genomics*, **7**, 64. doi:10.1186/s12920-014-0064-y.

[29] Brownstein, C.A. *et al.* (2014) An international effort towards developing standards for best practices in analysis, interpretation and reporting of clinical genome sequencing results in the CLARITY Challenge. *Genome Biology*, **15**, 3, R53. doi: 10.1186/gb-2014-15-3-r53.

[30] Lee, S. *et al.* (2014) Rare-Variant Association Analysis: Study Designs and Statistical Tests. *American Journal of Human Genetics*, **95**, 1, 5–23. doi:10.1016/j.ajhg.2014.06.009.

[31] Fromer, M. and Purcell, S.M. (2014) Using XHMM Software to Detect Copy Number Variation in Whole-Exome Sequencing Data. *Current Protocols in Human Genetics / Editorial Board, Jonathan L. Haines ... [et Al.]*, **81**, 7.23.1–7.23.21. doi:10.1002/0471142905.hg0723s81.

[32] Krumm, N. *et al.* (2012) Copy number variation detection and genotyping from exome sequence data. *Genome Research*, **22**, 8, 1525–1532. doi:10.1101/gr.138115.112.

[33] Do, R. *et al.* (2012) Exome sequencing and complex disease: practical aspects of rare variant association studies. *Human Molecular Genetics*, **21**, R1, R1–9. doi: 10.1093/hmg/dds387.

[34] Sundstrom, D. (2015). Even faster: Data at the speed of Presto ORC.

[35] Lipcon, T., Alves, D., Burkert, D., Cryans, J., Dembo, A., Percy, M., Rus, S., Wang, D., Bertozzi, M., McCabe, C.P. *et al.* (2015). Kudu: Storage for Fast Analytics on Fast Data.

[36] Dean, J. and Ghemawat, S. (2004) MapReduce: Simplified Data Processing on Large Clusters. *To appear in OSDI*, p. 1.

[37] Zaharia, M., Chowdhury, M., Das, T., Dave, A., Ma, J., McCauley, M., Franklin, M.J., Shenker, S. and Stoica, I. (2012) Resilient distributed datasets: A fault-tolerant abstraction for in-memory cluster computing. In *Proceedings of the 9th USENIX conference on Networked Systems Design and Implementation.* USENIX Association, pp. 2–2.

[38] Chaudhuri, S. and Dayal, U. (1997) An overview of data warehousing and OLAP technology. *ACM Sigmod record*, **26**, 1, 65–74.

[39] Cornell, M., Paton, N.W., Wu, S., Goble, C., Miller, C.J., Kirby, P., Eilbeck, K., Brass, A., Hayes, A., Oliver, S.G. *et al.* (2001) GIMS-a data warehouse for storage and analysis of genome sequence and functional data. In *Bioinformatics and Bioengineering Conference, 2001. Proceedings of the IEEE 2nd International Symposium on.* IEEE, pp. 15–22.

[40] Liu, X. *et al.* (2015) dbNSFP v3.0: A One-Stop Database of Functional Predictions and Annotations for Human Non-synonymous and Splice Site SNVs. *Human Mutation.* doi:10.1002/humu.22932.
